# Female reproductive tract pain: targets, challenges, and outcomes

**DOI:** 10.3389/fphar.2014.00017

**Published:** 2014-02-13

**Authors:** Phillip Jobling, Kate O’Hara, Susan Hua

**Affiliations:** School of Biomedical Sciences and Pharmacy, The University of Newcastle, CallaghanNSW, Australia

**Keywords:** pelvic pain, vagina, cervix, uterus, drug delivery

## Abstract

Pain from the female reproductive tract (FRT) is a significant clinical problem for which there are few effective therapies. The complex neuroanatomy of pelvic organs not only makes diagnosis of pelvic pain disorders difficult but represents a challenge to development of targeted therapies. A number of potential therapeutic targets have been identified on sensory neurons supplying the FRT but our knowledge on the basic neurophysiology of these neurons is limited compared with other viscera. Until this is addressed we can only guess if the new experimental therapies proposed for somatic, gastrointestinal, or bladder pain will translate to the FRT. Once suitable therapeutic targets become clear, the next challenge is drug delivery. The FRT represents a promising system for topical drug delivery that could be tailored to act locally or systemically depending on formulation. Development of these therapies and their delivery systems will need to be done in concert with more robust *in vivo* and *in vitro* models of FRT pain.

## INTRODUCTION

Pain syndromes represent one of the major challenges of neurology. Pain has many definitions but essentially it is a concept generated across the brain in response to internal or external stimuli that the individual associates with real or perceived tissue damage or imminent threat (Merskey and Bogduk, 1994). Pain is difficult enough to treat when it arises from a relatively straightforward injury to a defined region like a small piece of skin or a single joint. Pain from pelvic organs, particularly the reproductive tract, is notoriously difficult to treat. In this review we will examine the complex and unique innervation of the female reproductive tract (FRT), current treatments and the potential for topical therapies.

The prevalence of transient pelvic pain (usually dysmenorrhea) has been placed as high as 70–80% of women surveyed while chronic pelvic pain was reported at >20% (Hillen et al., 1999; Pitts et al., 2008). Ten percent of outpatient gynecological visits are for intractable pelvic pain (Ryder, 1996), and pelvic pain is the primary reason for 12–18% of hysterectomies (Kramer and Reiter, 1997). United kingdom estimates from 2000 placed direct healthcare costs at £158 million (Stones et al., 2000) whereas 1996 data from the USA placed patients’ out of pocket expenses at $1.9 billion dollars and indirect costs due to time off work at over $500 million (Mathias et al., 1996). Importantly many women do not seek treatment for their pain (Mathias et al., 1996).

Chronic pelvic pain is further divided into “specific disease-associated pelvic pain” and “chronic pelvic pain syndrome” where the underlying pathology remains obscure (International Association for the Study of Pain, 2011). Pelvic pain may arise from a number of structures, both somatic (e.g., striated pelvic floor muscles), and visceral (reproductive tract, bladder, and lower bowel). Focusing on reproductive structures, clinical observations have identified numerous predictors of chronic pelvic pain including endometriosis, pelvic inflammatory disease, childbirth, and urinogenital atrophy following menopause (Giamberardino, 2008; Lara et al., 2009; Paterson et al., 2009).

## THE COMPLEX NATURE OF PAIN FROM THE FRT

Sexual behavior and reproduction rely on the integration of nervous and hormonal signals to a widely distributed collection of structures. The external genitalia are essentially somatic structures and the distribution of sensory axons and their neurochemical coding are similar to cutaneous tissues (Martin-Alguacil et al., 2008; Moszkowicz et al., 2011; Vilimas et al., 2011). Sensory neurons innervating the hollow organs show different patterns of neurochemical expression compared with those that supply somatic structures (skin, muscle, and joints; Cervero and Laird, 2004; Song et al., 2009) and marked differences in central axons termination in the spinal cord (Sugiura et al., 1989, 1993).

Pain arising from the vagina, cervix, and uterus is an example of visceral nociception – or pain that comes from distension, injury, or inflammation of hollow organs (Cervero and Laird, 1999). Visceral pain is diffuse, poorly localized, often referred to other body regions, and can be accompanied by disrupted motor and autonomic reflexes (Janig and Morrison, 1986; McMahon, 1997; Westlund, 2000).

### EXTERNAL GENITALIA

**FIGURE 1:**
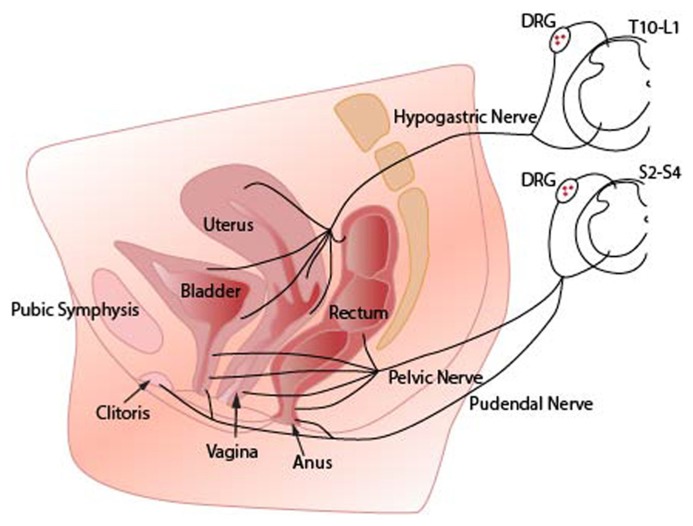
**Innervation of pelvic organs.** Sensory axons innervating the vagina reach the spinal cord via pelvic nerves and terminate in sacral spinal cord segments (S2–S4). Axons innervating the uterus travel in the hypogastric nerves and terminate in the thoracolumbar spinal cord segments (T10–L2). The region surrounding the cervix represents a transitional zone and is innervated by fibers that travel in both nerves. Sensory axons from the clitoris and vulva follow the pudendal nerves to sacral spinal cord. Note that sensory information from all pelvic organs may converge onto the same spinal cord neural circuits. DRG (dorsal root ganglia).

The most widely reported pain syndromes associated with external genitalia are the vulvodynias (Petersen et al., 2008; Fugl-Meyer et al., 2012). These have a prevalence of around 10% in U.S studies (Harlow and Stewart, 2003; Petersen et al., 2008). Most sensations from the external genitalia are transmitted via axons in the pudendal nerve (Martin-Alguacil et al., 2008; Moszkowicz et al., 2011; Vilimas et al., 2011; **Figure 1**). Limited data indicate that often pain from these structures is similar to generalized somatic pain as opposed to visceral pain (Bachmann et al., 2006; Goldstein and Burrows, 2008).

### SENSATIONS FROM THE VAGINA, CERVIX AND UTERUS ENTER THE CNS AT MULTIPLE LEVELS

The walls of the FRT are innervated with sensory afferent terminals that respond to both distension and inflammatory mediators (Berkley et al., 1993b; Papka and Traurig, 1993). The FRT is innervated via two main spinal nerve trunks; the hypogastric and pelvic nerves that send sensory information to a number of spinal cord segments (**Figure 1**; Berkley et al., 1993a,b; Wesselmann and Lai, 1997; Wesselmann, 2001; Jobling et al., 2003, 2010).

Electrical recordings from axons in rodents indicate that there is a heterogeneous distribution of receptors. Most axons respond to distension, whilst others respond to both distension and chemical stimuli (e.g., bradykinin or serotonin; Berkley et al., 1993a,b). Compared with the FRT, sensory axons supplying the gastrointestinal tract (GIT) and bladder have been better studied. Five functional classes of sensory axons have been described in the GIT (Page et al., 2002; Brierley et al., 2004; Song et al., 2009) and four functional classes of bladder sensory axons have been identified (Zagorodnyuk et al., 2007). There is an extensive interaction between vagina, cervix, uterus, and somatic structures (Hotta et al., 1999) where there is considerable convergence of these pathways in the spinal cord. The most widely and long-recognized consequence of this convergence is referred pain (Head, 1893).

## CENTRAL SENSITIZATION “PELVIC PAIN WITHOUT PELVIC ORGANS”

A significant barrier to treatment is the observation that pelvic pain can exist in the absence of any obvious pathology. In fact pelvic pain is often resistant to the removal of the allegedly offending organs (Baskin and Tanagho, 1992). This observation is thought to be caused by the phenomenon of central sensitization. The mechanisms underlying central sensitization for somatic afferents have been examined in detail (Millan, 1999; Jones and Sorkin, 2003; Lu et al., 2009), however, central sensitization from FRT afferents remain poorly understood. Inflammation of the rat uterus increased receptive field size, and decreased thresholds for cervix afferents (Berkley et al., 1993a). Another study has shown that FRT inflammation recruits large numbers of neurons in the dorsal horn (Wesselmann et al., 2000).

## PELVIC ORGAN CROSSTALK – LINKING VAGINA, CERVIX, UTERUS BLADDER, AND BOWEL

Epidemiological data suggest strong comorbidity between inflammatory bowel disorders, interstitial cystitis, and pelvic pain (Whorwell et al., 1986). Some of this comorbidity might be explained by the sensory and motor pathways that link FRT, bladder, and lower bowel (Winnard et al., 2006; Klumpp and Rudick, 2008). Sensations from these organs share synaptic circuits in the spinal cord (Wyndaele et al., 2013) and may even share individual sensory neurons (**Figure 1**; Christianson et al., 2007). These functional and anatomical interactions have implications for the effectiveness of local topical therapies designed to act on one organ only.

## OVARIAN HORMONES ALTER SENSORY INNERVATION AND PAIN THRESHOLDS

Fluctuations in levels of ovarian hormones, particularly estrogen, are associated with changes in sensation, including pain, in a variety of tissues (Martin, 2009). This effect of estrogen on pain responses is no doubt due to the widespread expression of estrogen receptors which are located, not only in the FRT, but also on primary sensory neurons, spinal cord neurons, and higher brain centers (Papka et al., 2001; Papka and Mowa, 2003; Vanderhorst et al., 2009; Takanami et al., 2010). Notably, estrogen receptors are particularly concentrated in sacral spinal cord segments that are crucial to the control of pelvic organs (Vanderhorst et al., 2009). The role of estrogen in modulation of the nervous system is unclear (Balthazart and Ball, 2006). Estrogen can influence several receptors and ion channels in peripheral, spinal and supraspinal pathways. For example transient receptor potential (TRP) channels on primary afferent neurons are inhibited by activation of the beta subtype estrogen receptor (ERβ; Xu et al., 2008).

### OVARIAN HORMONE WITHDRAWAL ALTERS FRT AND CUTANEOUS SENSITIVITY

In humans menopause is associated with a drastic decrease in levels of ovarian hormones (Martin, 2009). With this altered hormonal status many women have increased pain from the FRT, especially the vagina, and some somatic tissues (Fillingim and Edwards, 2001; Samsioe, 2007; Martin, 2009). This post-menopausal vaginal hyperalgesia, typically presents as pain during intercourse (dyspareunia; Davis et al., 2005; Mac Bride et al., 2010). Various explanations have been proposed including vaginal atrophy (Forsberg, 1995). However the severity of pain is only loosely correlated with vaginal wall thickness (Kao et al., 2008), suggesting other factors are critical in this condition. Many of the painful urinogenital symptoms can be reversed by conventional systemic estrogen replacement although an increasing alternative is the use of local intravaginal estrogen replacement (Mac Bride et al., 2010).

## PERIPHERAL THERAPEUTIC TARGETS

Precise information about the nature of primary sensory afferent endings in uterus, cervix, and vagina is scant compared with somatic (Woolf and Ma, 2007) or other visceral targets (Blackshaw et al., 2007). Anatomical data from animals (Papka et al., 1985, 1995, 1999; Shew et al., 1991; Collins et al., 2002) and, rarely, humans (Fried et al., 1990; Bokor et al., 2009; Malvasi et al., 2010) suggest they express the same neurochemical markers and receptors as nearby viscera, e.g., bladder and bowel.

### OPIOIDS

Enkephalin immunoreactive axons in uterus and vagina have been reported in some mammals (Lakomy et al., 1994; Skobowiat et al., 2009), while mu and delta opioid receptors are present in human and mouse myometrium (Zhu and Pintar, 1998; Fanning et al., 2013). Functional measures of peripheral opioid receptor activation are not well documented for FRT afferents. However in GIT (Armstrong et al., 2005; Page et al., 2008) and bladder (Su et al., 1997) mechanosensitive sensory axons are modulated by opioid receptor agonists.

### TRP CHANNELS

The TRP family of channels have been a focus of somatic pain research for some time. TRPV1 channels are present on presumed nociceptive axons in rat vagina (Liao and Smith, 2011) and human cervix (Tingåker et al., 2008). Interestingly these have been proposed to underlie some of the adverse side effects of clotrimazole, an anti-mycotic agent (Meseguer et al., 2008). Furthermore estrogen amplifies pain evoked by uterine distension, via a TRPV1 receptor dependent mechanism (Yan et al., 2007). Information on other TRP channels (e.g., TRPM8 and TRPA1) is limited in the FRT although they are expressed on sensory nerves supplying the GIT (Blackshaw et al., 2010).

### TROPHIC FACTORS

Various growth factors particularly nerve growth factor (NGF) and members of the glial cell line-derived neurotrophic factor family of ligands are implicated not only in survival of some sensory neurons but their receptors have been suggested as targets for alleviating neuropathic pain (Koltzenburg et al., 1999; Boucher et al., 2000; Malin et al., 2006; Malin and Davis, 2008). Within the FRT NGF has been reported in the uterus (Lobos et al., 2005) and cervix (Chalar et al., 2003) and neurturin mRNA has been found in uterus (Widenfalk et al., 2000). Sensory neurons innervating the uterus were shown to express tyrosine receptor kinase A receptors (Chalar et al., 2003). Whether growth factors or their receptors modulate sensory afferent neurons from the FRT is unknown although they are implicated in bladder signaling (Klinger and Vizzard, 2008; Schnegelsberg et al., 2010).

### P2X RECEPTORS

ATP was implicated in pain signaling nearly four decades ago (Bleehen and Keele, 1977). The subsequent discovery of P2X receptors on sensory nerves (Cook et al., 1997) had led to much work on identifying subtypes of P2X receptors as therapeutic targets (North and Jarvis, 2013). Detailed studies of the FRT are lacking however P2X receptors have been identified on both uterine and cervical sensory axons (Papka et al., 2005).

### METABOTROPIC GLUTAMATE RECEPTORS (mGluRs)

mGluR have been implicated in uterine and cervix sensory signaling (Ghosh et al., 2007) where they may modulate sensory discharge during parturition. Within the GIT peripheral mGluR on sensory endings modulate excitability (Page et al., 2005) where they have been proposed as therapeutic targets (Blackshaw et al., 2011).

### ACID SENSING ION CHANNELS (ASICs)

No studies to date have tested whether ASIC are expressed on sensory neurons innervating the FRT. However, they are expressed in vagal (Page et al., 2007) and colonic (Jones et al., 2005) sensory neurons where they represent a potential therapeutic target.

### NITRIC OXIDE

Nitric oxide generated by neuronal, inducible or endothelial nitric oxide synthase (NOS) plays many roles in the FRT. It is most notably released by autonomic vasodilator neurons to dramatically increase blood flow (Morris et al., 2005). However nNOS is also expressed in a subpopulation of sensory nerves (Papka et al., 1995). The role of nNOS in sensory signaling in the reproductive tract is unknown. However a role for pain modulation has been proposed in somatic pain models (Boettger et al., 2007; Keilhoff et al., 2013) and therapeutic agents that target nNOS have been proposed (Mladenova et al., 2012).

### CANNABINOIDS

The cannabinoid signaling pathways have long been proposed as therapeutic targets (Roques et al., 2012). The FRT has some of the highest levels of endogenous cannabinoids (Schmid et al., 1997) and cannabinoid receptors are expressed in human and rodent myometrium (Das et al., 1995; Dennedy et al., 2004) where they act on smooth muscle. Cannabinoid receptors on sensory axons associated with the FRT have not been reported. However activation of cannabinoid type 1 receptors modulates sensory afferent signaling from the urinary bladder (Walczak et al., 2009) and jejunum (Yuce et al., 2010).

## CURRENT THERAPIES FOR FRT PAIN

Currently evidence based treatment for FRT pain is limited. Standard pain therapies such as paracetamol, non-steroidal anti-inflammatory drugs (NSAIDs), opioids, or neuropathic pain therapies have been used, as the type of pain is not well defined and directing treatment is difficult. Topical therapies are increasing as options for treating vulvar and vaginal pain and are recommended as first line treatment (Nunns et al., 2010). Topical therapy presents an attractive alternative to systemic therapy as they are generally well tolerated and are associated with less systemic adverse effects. However, some topical therapies may cause irritation that can worsen symptoms (Nunns et al., 2010). Currently, due to a lack of evidence of effectiveness, all topical therapies used in this condition would be considered experimental (Andrews, 2011). Topical treatments have also been associated with a high placebo response (Nunns et al., 2010).

Vulvodynia and vestibulodynia treatment has been the subject of several recent reviews, which concluded there was insufficient evidence of effectiveness and safety for a range of therapies. It was determined there was evidence of a lack of efficacy for botulinum toxin injection, topical 5% xylocaine, and topical nifedipine. There was insufficient evidence to evaluate the effectiveness of steroid, local anesthetic injections, nerve blocks, intramuscular or intralesional interferon or topical capsaicin, montelukast, steroids, gabapentin, and ketoconazole (Andrews, 2011). Oral treatments that include tricyclic antidepressants, serotonin–norepinephrine uptake inhibitors and anticonvulsants (Cox and Neville, 2012) lack good quality evidence of effectiveness and have systemic adverse effects. Physical and alternative therapies are also used but there are only anecdotal reports of effectiveness (Andrews, 2011; Cox and Neville, 2012). Surgery has also been used effectively to treat vulvar vestibular pain (Nunns et al., 2010; Andrews, 2011; Cox and Neville, 2012). Estrogen therapies are effective in patients where the pain is linked to low estrogen levels following menopause or breast cancer treatment (Goetsch, 2012). Less common therapeutic options that have shown success in small clinical trials include cutaneous fibroblast lysate cream (Donders and Bellen, 2012), nitroglycerin cream (Walsh et al., 2002), and amitriptyline-baclofen cream (Nyirjesy et al., 2009).

Treatment of uterine pain is limited to systemic options with little evidence. Dysmenorrhea is the best-studied uterine pain syndrome. Primary dysmenorrhea is treated with simple analgesics, usually naproxen, while secondary dysmenorrhea treatment relies on removal of the underlying cause of the pain (Kohle and Deb, 2011). As other pelvic organs can cause pelvic pain a thorough investigation is important. Non-pharmacological therapies including nutrition and lifestyle changes, along with surgery may play a role. Further investigation is needed to determine the most appropriate treatments for uterine pain.

Further investigation is needed to determine specific targets for pharmacological management of the various FRT pain sub-types. The use of drug delivery systems may be required to effectively deliver existing or experimental compounds to the target site for improved efficacy and/or to reduce systemic adverse effects.

## DELIVERING THERAPIES TO THE FRT

The intravaginal route of drug administration has been studied as a suitable site for local and systemic delivery of therapeutic agents. The degree to which therapies act locally or systemically is formulation dependent. Presently intravaginal therapies are typically prescribed for vaginal infections and vaginal dryness. Systemic drug delivery includes uterine targeting or treatment of migraines (Bassi and Kaur, 2012). In relation to pain stemming from the FRT, the intravaginal route shows promise for the local or systemic delivery of analgesic and anti-inflammatory agents.

The vagina has unique features that can be exploited for optimal therapeutic responses, such as the presence of a dense network of blood vessels, large surface area, and permeability (Srikrishna and Cardozo, 2013). In addition, unlike conventional oral therapy, the vaginal route avoids hepatic first-pass metabolism, significant enzymatic degradation of active ingredients and drug interactions (Bassi and Kaur, 2012). Absorption from vaginal delivery systems occurs by dissolution, followed by penetration of drug through the vaginal membrane to reach the systemic circulation (Hussain and Ahsan, 2005). Physiological factors can affect the drug release from intravaginal delivery systems and/or vaginal absorption of drugs, such as cyclic changes in thickness of the vaginal epithelium, fluid volume and composition, pH and sexual arousal (Hussain and Ahsan, 2005; das Neves et al., 2011). Although physiological factors are difficult to alter, the physicochemical properties of a drug compound (e.g., molecular weight, lipophilicity, ionization, surface charge, chemical nature; Hussain and Ahsan, 2005) as well as the formulation can be selected to regulate local versus systemic activity.

Despite its therapeutic potential, vaginal preparations show low patient acceptability due to factors including multiple daily dosing; leakage and messiness following application; and the need for night-time dosing. The effectiveness of commonly available vaginal dosage forms (creams, gels, solutions, foams, pessaries) is often limited by their low retention to the vaginal epithelium (Pavelić et al., 2004). In order to overcome these limitations, novel vaginal delivery systems are being developed that possess desirable distribution, bioadhesion, and release properties – such as vaginal rings, bioadhesive delivery systems, and nanosystems.

### VAGINAL RINGS

Intravaginal rings (IVRs) are circular drug delivery devices that are designed to provide both sustained and controlled drug release, lasting for several weeks to several months following insertion into the vagina. IVR have been shown to be effective in delivering a multitude of compounds, such as contraceptive steroids and steroids for the treatment of post-menopausal atrophy. This delivery device has been previously reviewed (Baloglu et al., 2009; Thurman et al., 2013; Srikrishna and Cardozo, 2013).

### BIOADHESIVE DRUG DELIVERY (BDD) SYSTEMS

BDD systems were developed to circumvent the issues associated with conventional vaginal formulations, by adhering to the vaginal mucosal tissue and prolonging the residence time of the formulation. Vaginal BDD systems have been exploited for both local as well as systemic delivery of drugs (Merabet et al., 2005; Bassi and Kaur, 2012). Several studies have focused on BDD systems in the form of tablets, films, patches, and gels for the vaginal mucosal route that are composed of bioadhesive polymers that are biocompatible, biodegradable and stable. Common mucoadhesive polymers include tragacanth (acacia), carbopol resins, sodium alginate, carboxymethylcellulose, and chitosan. Vaginal BDD systems have been previously reviewed (Baloglu et al., 2009; Bassi and Kaur, 2012).

### NANOSYSTEMS

Nanocarriers [e.g., dendrimers, liposomes, Poly(lactic-co-glycolic acid) nanoparticles, silver and gold nanoparticles] have been utilized in topical drug delivery to enhance the penetration of drug compounds. For example, encapsulation of drugs within liposomes can provide characteristics such as enhanced skin or mucosal permeability, sustained release as well as controlled release (Pavelić et al., 2004). Such nanosystems are usually incorporated within a bioadhesive base (e.g., Carbopol resin) to enhance the viscosity of the formulation for retention on the mucosal surface (Pavelić et al., 2004; das Neves et al., 2011). The use of nanosystems is promising for intravaginal drug delivery, however, similar to BDD systems, the interaction of these formulations with mucosal fluids present in the vagina at different stages of the menstrual cycle and age is not yet well defined (Bassi and Kaur, 2012).

## CONCLUSION

Pain attributed to the FRT is complex and involves several classes of nociceptive and non-nociceptive sensory neurons. The unique neural anatomy of pelvic organs provides challenges in the delivery of selective therapies. There is little evidence that current treatments are effective and new strategies need to be developed. Relative to somatic pain, or pain from the GIT, there is a lack of information on the basic neurophysiology of FRT sensory neurons. Well defined animal models of neuropathic or inflammatory pain exist for somatic structures (e.g., chronic constriction injury models) and to some extent colitis (trinitrobenzene sulfonic acid models). At present there is no consistent approach to FRT pain. This will need to be addressed if we are to explore the many potential therapeutic targets present on FRT sensory neurons. Exciting opportunities exist for development of intravaginal drug delivery systems for either local or systemic drug delivery. Similar targeted delivery systems can be developed for the vulvodynias. Finally larger clinical trials of the few currently available promising therapies could provide useful insights in directing preclinical studies.

## Conflict of Interest Statement

The authors declare that the research was conducted in the absence of any commercial or financial relationships that could be construed as a potential conflict of interest.
